# Analysis of Characteristics in the Macro-Composition and Volatile Compounds of Understory Xiaobai White Tea

**DOI:** 10.3390/plants12244102

**Published:** 2023-12-07

**Authors:** Mengcong Zhang, Chengzhe Zhou, Cheng Zhang, Kai Xu, Li Lu, Linjie Huang, Lixuan Zhang, Huang Li, Xuefang Zhu, Zhongxiong Lai, Yuqiong Guo

**Affiliations:** 1 College of Horticulture, Fujian Agriculture and Forestry University, Fuzhou 350002, China; zmc.1998@foxmail.com (M.Z.); chengzhechou@foxmail.com (C.Z.); zhangcheng9742@foxmail.com (C.Z.); xukai97@foxmail.com (K.X.); lu18286159614@163.com (L.L.); huanglinjie1997@foxmail.com (L.H.); m15160004025@163.com (L.Z.); 13984128485@163.com (H.L.); laizx01@163.com (Z.L.); 2 Institute of Horticultural Biotechnology, Fujian Agriculture and Forestry University, Fuzhou 350002, China; 3 Nanping Jianyang District Tea Development Center, Nanping 353000, China; 18950687671@163.com; 4 Anxi College of Tea Science (College of Digital Economy), Fujian Agriculture and Forestry University, Quanzhou 362400, China; 5 Tea Industry Research Institute, Fujian Agriculture and Forestry University, Fuzhou 350002, China

**Keywords:** understory planting, environment factors, fresh-leaf, Xiaobai white tea, volatile compounds

## Abstract

Understory planting affects the growth environment of tea plants, regulating the tea plant growth and the formation of secondary metabolites, which in turn affects the flavor of Xiaobai white tea. The present research adopted biochemical composition determination, widely targeted volatilities (WTV) analysis, multivariate statistical analysis, and odor activity value (OAV) analysis to analyze the characteristics in the macro-composition and volatile compounds of understory white tea. The sensory evaluation results indicated that understory Xiaobai white tea (LWTs) was stronger than ordinary Xiaobai white tea (PWTs) in terms of the taste of smoothness, sweetness, and thickness as well as the aromas of the flower and sweet. Understory planting reduced light intensity and air temperature, increased air humidity, organic matter, total nitrogen, and available nitrogen contents, which improved the growth environment of tea plants. The phytochemical analysis showed that the water-extractable substances, caffeine, flavonoids, and soluble sugar contents of understory tea fresh-leaf (LF) were higher than those of ordinary fresh-leaf (PF). The phytochemical analysis showed that the free amino acids, theaflavins, thearubigins, water-extractable substances, and tea polyphenols contents of LWTs were significantly higher than those of PWTs, which may explain the higher smoothness, sweetness, and thickness scores of LWTs than those of PWTs. The 2-heptanol, 2-decane, damasone, and cedar alcohol contents were significantly higher in LWTs than in PWTs, which may result in stronger flowery and sweet aromas in LWTs than in PWTs. These results provide a firm experimental basis for the observed differences in the flavor of LWTs and PWTs.

## 1. Introduction

Tea is a shade plant, adapted to the understory of forests in its native habitat [1]. White tea is one of six kinds of tea in China and undergoes the fewest manufacturing processes, including prolonged withering and drying [2]. According to the different cultivars of tea trees from which fresh leaves are derived, white tea can be divided into Dabai, Xiaobai, and Shuixian white tea. Xiaobai white tea is one of the high-quality white teas in the Fujian Province, has a long history, with a slightly sweet and umami taste as well as a fresh odor, widely loved by consumers. The quality of Xiaobai white tea is determined by the fresh-leaf quality and the tea-making process, fresh-leaf is the foundation. Environmental factors, such as temperature, humidity, light, and rainfall were closely related to the growth and development of tea plants and the accumulation of fresh leaf components [3]. Tea plants grow well in conditions of 80% relative humidity and cloudy conditions (sunshine percentage ≤40%) [4,5]. Suitable effective accumulated temperatures (112.4 °C, with a minimum daily temperature of 5 °C), large temperature changes between the day and night, and scattered light, which contributes to the accumulation of aroma substances in tea, will increase the freshness and taste of tea to a certain extent, resulting in high-quality green tea [6]. Tea plants is an acidophilic plant, and the soil pH of 4.5~5.5 is suitable for its cultivation [7]. Soil type affects the exchange of water, air, and nutrients between the soil and the tea plant, which ultimately affects the growth and yield of the tea plant [8]. Nitrogen (N), phosphorus (P), and potassium (K) are the three main nutrients that regulate the growth of tea plants, and their availability directly affects the yield and quality of tea [9]. N and P are mainly involved in the synthesis of amino acids, vitamins, chlorophyll, and other substances, and directly regulate the content and proportion of biochemical components of the tea plants [10,11]. K helps to promote the growth and development of tea seedlings and the absorption capacity of tea roots, thus enhancing the resilience of tea plants [3].

Agroforestry system is a form of understory planting that involves planting trees alongside crops to improve the capture of temporal resources between species [12,13], thereby increasing resource use efficiency. Historically, agroforestry systems have been widely implemented as a way to achieve sustainability in agriculture [14,15]. To reduce the required labor force and protect the ecological environment, many places including Chongqing, Sichuan, Hubei, and Anhui planted Coptis chinensis Franch under natural forests using natural shade trees [16]. To overcome farmland shortage, understory *P. notoginseng* was developed as a new ecological planting model [17]. A related study investigated the production patterns of black oats grown under four different woodland types in southern Brazil and found that black oats grown under *P. rigida* were the most productive [18]. Agroforestry systems have been successfully practiced and researched on many species. The agroforestry pattern is also widely used in tea, many areas in Fujian Province have tried to plant tea under the forest. Using the existing forest land, the forest land is thinned with care without changing the stand structure, and tea plants are planted in the forest. Compared with ordinary planting methods, shade planting under the forest significantly changes the growing environment of tea. The chlorophyll content in the new shoots of tea plants in spring, summer, and autumn increased to varying degrees after shade tea plants [19]. Shao et al. [4] found in the study of the difference of metabolites processed into different tea types by preharvest shading that shading had a great effect on the composition of amino acids, flavonoids, and theaflavins in tea. The thickening of fence tissue and thinning of sponge tissue in tea fresh leaf cells in a shaded environment contributed to the accumulation of volatile metabolites, including volatile fatty acid derivatives and volatile phenylpropyl/phenyl esters (VPBs) [20,21], the accumulation may be related to upstream metabolism. Fang et al. [22] showed that preharvest shade covering helped to increase the total volatile compounds of steamed green tea. Understorey planting of tea plants improved soil organic matter, total nitrogen, total phosphorus, and total potassium content compared to ordinary tea plant gardens [23], which in turn improved the quality of tea plants.

To explore the influence of understory planting on the flavor quality of Xiaobai white tea, this study measured the main environmental factors of the understory Xiaobai white tea plantation and one ordinary Xiaobai white tea plantation. Then, the fresh leaves of the understory Xiaobai white tea plantation and ordinary Xiaobai white tea plantation were taken and processed into dry tea as experimental materials. The wide range of targeted volatile metabolomics (WTV) method, and odor activity values (OAV) method combined with stoichiometry were used to explore the effects of understory planting on the growth environment and fresh-leaf components of tea trees. Then screen the quality difference between LWTs and PWTs, which was aimed at providing a firm experimental basis for the promotion of understory tea planting mode.

## 2. Results and Discussion

### 2.1. Understory Planting Improves the Growing Environment for Tea Plants

**Figure 1:**
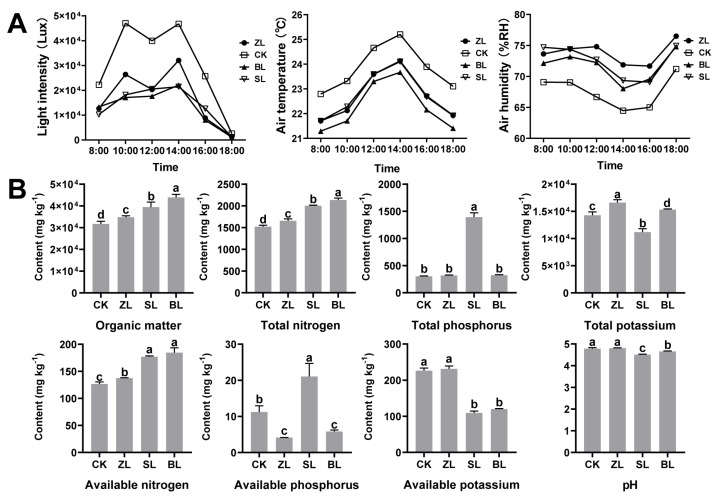
(**A**) Light intensity, air temperature, and air humidity, (**B**) the content of organic matter, total nitrogen, total phosphorus, total potassium, available nitrogen, available phosphorus, available potassium, and pH. The various superscripts show significant differences (*p* < 0.05).

Tea can achieve high quality only when it grows in the suitable environmental conditions of light temperature, humidity, soil, and fertilizer [3]. When the light intensity exceeds 3.7 J/cm^2^·min, the photosynthetic rate of tea leaves will decline, and the optimal temperature for the growth and development of tea new shoots is at 20–25 °C, more than this temperature range, the new shoots growth rate slows down [24]. Hot and dry weather tends to make green tea bitter and astringent [25]. As shown in Figure 1A, the light intensity in the ordinary Xiaobai white tea garden (CK) was roughly twice as high as under shading. The average air temperature under shading was 1.27 °C lower than that in CK, and the average air humidity under shading was 5.09% RH higher than that in CK, respectively. These indicate that understory planting improves the growth environment of tea plants and the quality of tea. In addition, the nutrient status of the soil also has an important impact on the quality of tea, 11.0% nitrogen (N), 1.65% phosphorous (P), and 3.7% potassium (K) are reported to be necessary nutrients for tea [26]. As shown in Figure 1B, the organic matter, total nitrogen, and available nitrogen contents under shading were significantly higher than those in CK, other factors did not show a uniform pattern in the shaded and unshaded groups. In line with previous studies, Wen et al. [27] found that the soil nutrient content of the three intercropping systems was higher than that of the tea monoculture system, except for phosphorus [28]. The composition of the soil nutrients in the intercropping system is affected by many factors, such as the combination of different plant species, plant residues, and vertical structure [29]. Different forests have different soil nutrient requirements, which may be the reason why factors such as total phosphorus and total potassium in *Castanea henryi* understory Xiaobai white tea plantation (ZL), *Cunninghamia lanceolata* understory Xiaobai white tea plantation (SL), and *Phyllostachys edulis* understory Xiaobai white tea plantation (BL) did not show consistent regularity. In short, understory planting improves the growing environment of tea plants.

### 2.2. Impact of Understory Planting on Fresh-Leaf Contents

**Figure 2:**
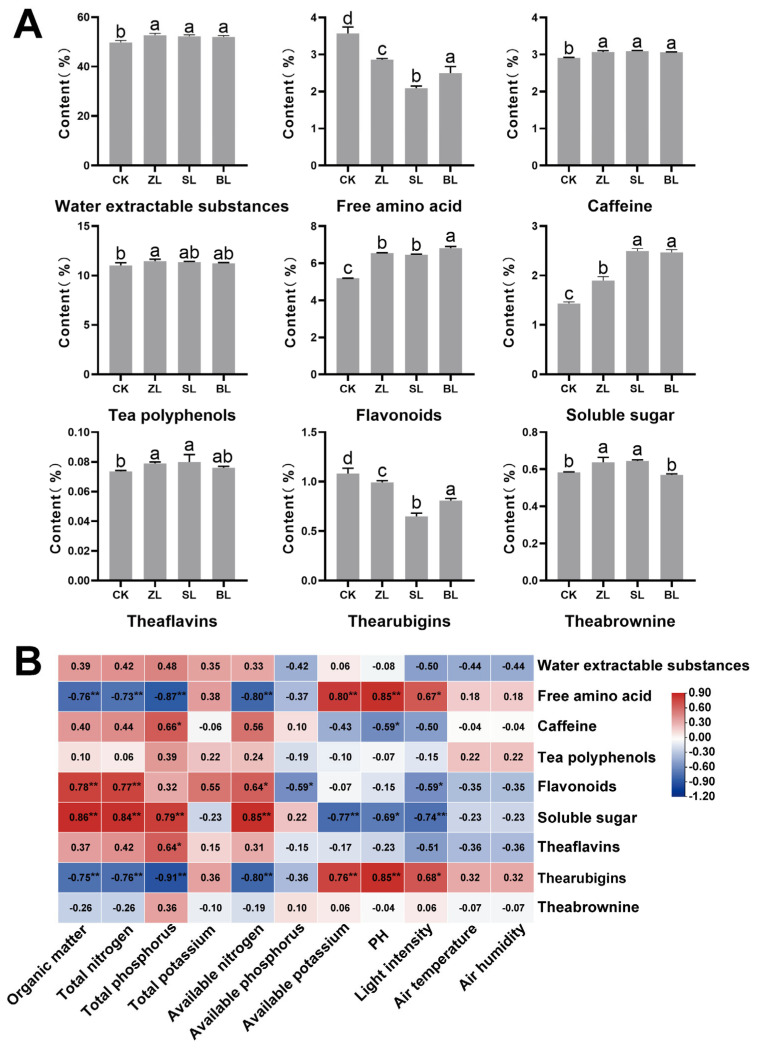
(**A**) The content of water-extractable substances, free amino acids, caffeine, tea polyphenols, flavonoids, soluble sugar, theaflavins, thearubigins, and theabrownines, (**B**) Correlation between macroscopic components and major environmental factors. The various superscripts show significant differences (*p* < 0.05). The symbols * and ** indicate statistical significance at *p* < 0.05 and *p* < 0.01, respectively. The sample information of fresh leaves is shown in Table 1.

To understand the effect of understory planting on the content of tea plants, the macroscopic components of tea fresh-leaf were determined and analyzed. The relative total water-extractable substances, caffeine, flavonoids, and soluble sugar contents in LF are significantly higher than those in PF, and the relative contents of free amino acids and thearubigins in LF are lower significantly than those in PF (Figure 2A); these provide a good foundation for the processing of dry tea. The growth of the tea plant is not only affected by its genetic factors, but also regulated by environmental factors such as light, temperature, water, and fertilizer, resulting in differences in the quantity and proportion of the components contained in the fresh-leaf [30]. Correlation analysis is a powerful tool for investigating the relationship between major environmental factors and the macroscopic components, the calculated results of the Pearson correlation coefficient between the two show that (Figure 2A), organic matter, total nitrogen, total phosphorus, available nitrogen, available phosphorus, available potassium, pH, and light intensity correlated closely with different macroscopic components (r > 0.6, *p* < 0.05), indicating that they are important factors underlying the macroscopic components of tea fresh-leaf.

Sponge tissue has a storage function, contains a large number of polyphenols and carbohydrates related to tea quality, and shading promotes the relative proportion of sponge tissue in leaves, which helps the accumulation of components contained in fresh-leaf [31]; this is consistent with the results of a negative correlation between water extract and light intensity (Figure 2B). The accumulation of free amino acid content in tea leaves under shade conditions is not due to increased synthesis but to the hydrolysis of chloroplast proteins [32]. Early shading can play a role in increasing the amino acid content of fresh leaves. This effect on the increase in amino acid content gradually weakens with the extension of shading time [33]; moreover, this is consistent with the fact that free amino acid have a significant negative correlation with organic matter, total nitrogen, and available nitrogen (Figure 1B and Figure 2B). There was a significant positive correlation between the soil pH and free amino acids, which indicated that a proper increase in the pH value could improve the tea quality. However, it is not advisable to increase the soil pH above six, as that would be outside the fitness range of the tea tree [34]. When the soil pH value is greater than six, the growth of tea tree shoots is inhibited to a greater extent than nitrogen uptake, which reduces the yield of tea [35]. In the previous study, the long-term shading remarkably inhibited sugar metabolisms such as glycolysis, galactose metabolism, and pentose phosphate pathway in the leaves and roots of “Xiangfeicui” [36] to replenish the carbon backbone during shading, plants hydrolyze large amounts of polysaccharides into soluble sugars [37], which corresponded well with our findings. There was a significant positive correlation between soluble sugar and total nitrogen and available nitrogen, which was consistent with the report that the soluble sugar content decreased due to long-term nitrogen fertilizer loss [38]. The results suggested that the low content of available nitrogen in the soil led to the weakening of carbon assimilation in tea plants [39].

### 2.3. Significant Difference in Aroma and Flavor between LWTs and PWTs

**Figure 3:**
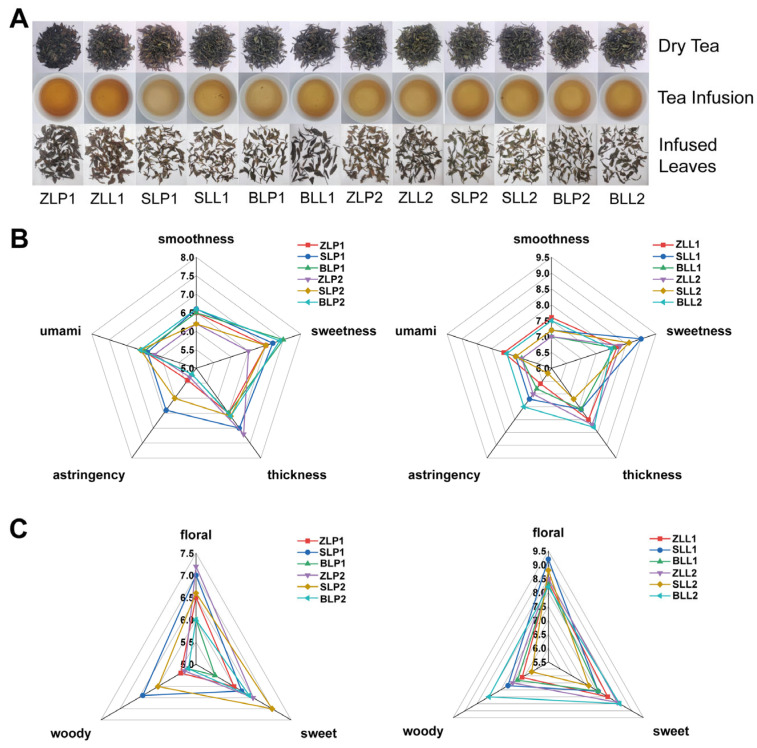
(**A**) The appearance and infusion colors of PWTs and LWTs; (**B**) Spider plots for the taste profiles of PWTs and LWTs; (**C**) Spider plots for the aroma profiles of PWTs and LWTs.

The quality of fresh tea leaves determines the quality of tea products. However, the quality-related metabolites in fresh leaves are significantly altered by the post-harvest processing of tea leaves. To more comprehensively analyze the effects of understory planting on the quality of tea products, also to enhance the representativeness of this study, repeated sampling was carried out. On 26 April, the *Castanea henryi* understory Xiaobai white tea samples (ZLL1) and the ordinary Xiaobai white tea samples (ZLP1) were obtained. On 30 April, the *Cunninghamia lanceolata* understory Xiaobai white tea samples (SLL1) and ordinary Xiaobai white tea (SLP1) were obtained. On 2 May, the *Phyllostachys edulis* understory Xiaobai white tea samples (BLL1) and ordinary Xiaobai white tea samples (BLP1) were obtained. On 7 May, 9 May, and 13 May, the *Castanea henryi* understory Xiaobai white tea samples (ZLL2) and ordinary Xiaobai white tea samples (ZLP2), the *Cunninghamia lanceolata* understory Xiaobai white tea samples (SLL2) and ordinary Xiaobai white tea samples (SLP2), the *Phyllostachys edulis* understory Xiaobai white tea samples (BLL2) and ordinary Xiaobai white tea samples (BLP2) were obtained again, respectively. Taken together, a total of 12 tea samples were obtained including ZLL1 and ZLP1, SLL1, and SLP1, BLL1, and BLP1, ZLL2, and ZLP2, SLL2, and SLP2, BLL2, and BLP2 (Table 2). All samples were processed in Zhangdun Town, Jianyang District, Nanping City using unified picking standards and processing techniques. The quality of the white tea was assessed according to the aroma and taste compounds and sensory properties. The sensory evaluation results showed that the appearance was in all samples, with the whole shoot and greenish auburn. In addition to ZL1, there were all LWTs in other groups, and PWTs in SL1 also had tippy. A yellow and bright liquor color was defined in all samples, the difference is that deeper of liquor color in LWTs than in PWTs (Figure 3A). LWTs were stronger than PWTs in terms of the taste of smoothness, sweetness, and thickness (Figure 3B), both LWTs and PWTs have a floral and sweet aroma, individual samples have a woody aroma, but the floral aroma of LWTs is stronger and more lasting than PWTs (Figure 3C). In general, LWTs has a more pleasant flavor than PWTs.

### 2.4. Analysis of Macro-Composition of LWTs and PWTs

**Figure 4:**
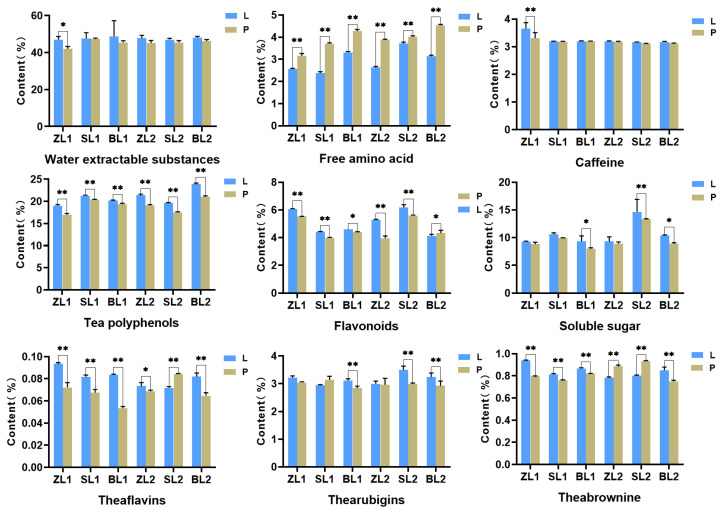
The content of water-extractable substances, free amino acids, caffeine, tea polyphenols, flavonoids, soluble sugar, theaflavins, thearubigins, and theabrownines. The symbols * and ** indicate statistical significance at *p* < 0.05 and *p* < 0.01, respectively. Undergrowth-picked was referred to as L, and ordinary-picked was called P.

The taste of tea is mainly determined by macro-composition, included among these, tea polyphenols enhance the bitterness of tea infusions, flavonoids supply the bitter taste, free amino acids are the source of the umami taste, soluble sugars are the main contributors to sweetness [40]. The relative total water-extractable substances, tea polyphenols, caffeine, and soluble sugar contents in LWTs are higher than those in PWTs and the relative contents of free amino acids contents in LWTs are lower than those in PWTs (Figure 4). This may be the reason for why LWTs has a stronger smoothness, sweetness, and thickness than PWTs in the taste. The macro-composition of fresh-leaf and tea are basically the same, this shows that the matrix of fresh-leaf contents contributes to the formation of good-quality tea. TRs, TBs, and TFs did not show uniform regular differences in the six groups of samples, which may be due to their unique woodland environment. During the withering process of white tea, on the one hand, the hydrolysis of large molecules within the withered leaves, starch, proteins, and other substances are hydrolyzed to monosaccharides, amino acids, and other water-soluble small molecules. On the other hand, along with the enhancement of the activity of enzymes such as polyphenol oxidase, the polyphenols, mainly catechins, undergo an oxidation and condensation reaction, which gives rise to oxidative products such as pigments and other products, and form the unique flavor of white tea.

**Figure 5:**
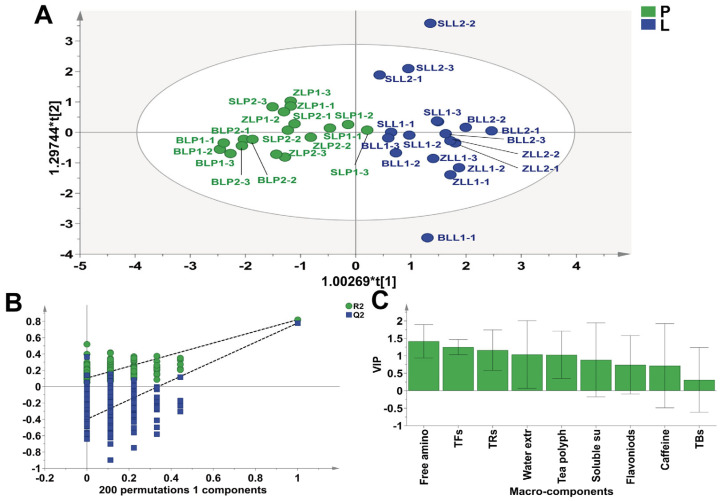
(**A**) The OPLS-DA scores for the non-volatile components; (**B**) Permutation test plots of the non-volatile components; (**C**) The variable importance in the project (VIP) of the non-volatile components. Undergrowth-picked was referred to as L, and ordinary-picked was called P.

To further identify the key macro-components that differentiate the tastes of LWTs and PWTs, orthogonal partial least squares–discriminant analysis (OPLS-DA) was performed on the macro-compositions (Figure 5A). Cross-validation analysis showed that the OPLS-DA models were reliable (Figure 5B). LWTs gathered in the first and fourth quadrants, and in PWTs, except for SLP1-3, the rest are clustered in the second and third quadrants (Figure 5A). The indication of the characteristic macro-composition helps to distinguish LWTs from PWTs. We can know from Figure 5C that the variable importance in projection (VIP) value of free amino acids, TFs, TRs, water-extractable substances, and tea polyphenols is greater than 1, and metabolites with VIP > 1 are generally considered to be significantly different. In the previous study [41], free amino acids, flavonoids, and TFs are the main metabolic pathways that improve the flavor of shade tea, which is basically consistent with this study.

### 2.5. Identification of VOCs in LWTs and PWTs

#### A Total of 220 Volatiles Were Detected

**Figure 6:**
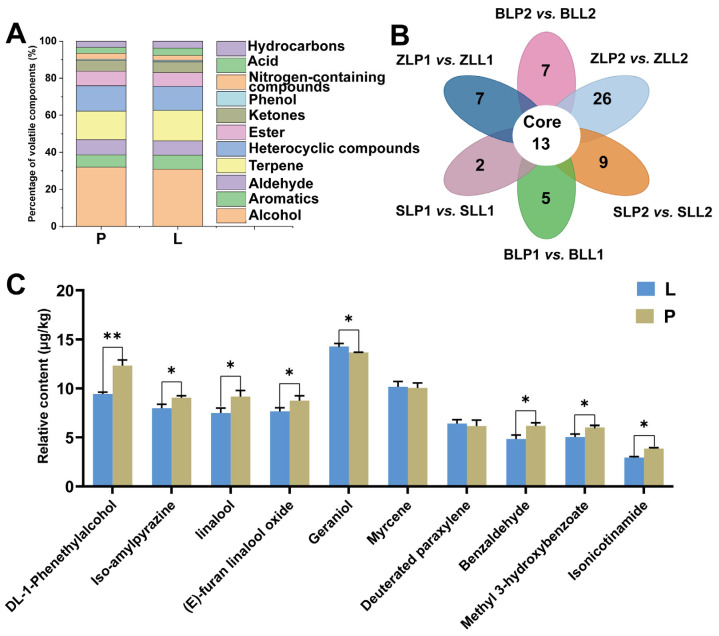
(**A**) The ratio of volatile components; (**B**) Venn diagram for different groups of key volatile components in BLP2 vs. BLL2, ZLP1 vs. ZLL1, ZLP2 vs. ZLL2, SLP1 vs. SLL1, SLP2 vs. SLL2, BLP1 vs. BLL1; (**C**) The content of top ten volatile components. Undergrowth-picked was referred to as L, and ordinary-picked was called P. The symbols * and ** indicate statistical significance at *p* < 0.05 and *p* < 0.01, respectively.

We used HS-SPME in combination with GC-MS/MS to determine the volatile content of LWTs and PWTs. The total ion flow (TIC) plot of the mixed sample is superimposed to determine the reproducibility of volatile extraction and detection, then the high stability of the instrument ensures data repeatability and reliability (Supplementary Figure S1). In total, 220 volatiles were detected, including 16 alcohols, 25 aromatic hydrocarbons, 8 phenols, 6 nitrogenous compounds, 15 aldehydes, 7 acids, 34 hydrocarbons, 28 ketones, 22 terpenes, 33 heterocyclic compounds, and 26 esters (Supplementary Table S1); the percentage of each substance is shown in the Figure 6A. Overall, the volatile metabolite composition in LWTs and PWTs is similar, but the exact content is slightly different (Supplementary Table S1). During the drying process, high temperatures produce the Maillard reaction, which causes amino acids and carbonyl compounds to polymerize and condense at room temperature or when heated, producing a large number of heterocyclic compounds [41,42], this may be the reason for the high content of volatile heterocyclic compounds in PWTs and LWTs.

Among all VOCs, the top ten substances with relative content are DL-1-Phenethylalcohol, iso-amylpyrazine, linalool, (E)-furan linalool oxide, geraniol, myrcene, deuterated paraxylene, benzaldehyde, methyl 3-hydroxybenzoate, and isonicotinamide (Figure 6C). The relative content of geraniol in LWTs was the highest, accounting for an average of 10.11%, followed by myrcene (7.21%), DL-1-Phenethylalcohol (6.83%), iso-amylpyrazine (5.62%), and (E)-furan linalool oxide (5.42%), mainly displayed floral and green. The highest relative content in PWTs is geraniol, accounting for an average of 9.15%, followed by threuterol (8.28%), myrcene (6.70%), linalool (6.13%), and iso-amylpyrazine (6.03%), mainly displayed floral. The differences in the composition of these VOCs and differences in relative content may be responsible for the difference in the aroma of LWTs and PWTs.

### 2.6. Screening for Differential VOCs in LWTs and PWTs

#### 2.6.1. Thirteen Important Differential VOCs with LWTs and PWTs Were Screened for

To investigate the VOCs responsible for the sensory differences between LWTs and PWTs, we used OPLS-DA further to analyze each of the six sets of samples. The OPLS-DA discriminant model is not overfitted, and the model is reliable, the LWTs and PWTs being significantly differentiated by VOCs (Supplementary Figure S2).

There were 52 (ZL1), 26 (SL1), 45 (BL1), 76 (ZL2), 55 (SL2), and 42 (BL2) compounds with VIP values greater than 1 and FC greater than 1.5 or FC less than 0.67 in the OPLS-DA model that was developed in this study. There are 13 important differential volatile metabolites shared by the six groups, namely 2-heptanol, cedrol, m-Xylene, β-isophorone, 2-Decanone, Jasmone, β-Damascenone, (+)-CUPARENE, (−)-Calamenene, 2-Aminopyridine, decyl formate, benzyl acetate, and cis-3-Hexenyl 2-methylbutanoate (Figure 6B), these are the important differentiating VOCs that distinguish LWTs from PWTs (Supplementary Table S2). Further analysis of the 13 VOCs showed that ketones compounds dominated the numbers, followed by esters. The highest VIP value (mean VIP value for the six groups of samples) was benzyl acetate, followed by decyl formate, (+)-CUPARENE, and (−)-Calamenene, which mainly display floral and herbal aromas. The benzyl acetate (jasmine, rose) relative content in LWTs (51.42 μg/kg on average, relative to internal standard) was almost twice that of the content in PWTs (98.61 μg/kg on average, relative to internal standard), which may contribute to a stronger flowery aroma for LWTs than PWTs.

Ketones generally have a “floral” and “woody” aroma [43]. In the previous study, the content of jasmone (jasmine) in tea was significantly affected by the different altitudes and seasons, at high altitudes with short sunshine hours and weak intensity, the content of jasmone in autumn tea is higher than that at low altitudes and summer [44], which is consistent with our study. Damasone, which has a flowery and fried potatoes aroma, was found to be the main contributor to the most to the aroma of Keemun black tea [45], and there is a downward trend with the increase in storage time in An tea [46]. 2-decane, which has a floral and citrus aroma, was found to be a characteristic volatile metabolite of beef bouillons. In the other study, 2-decone was produced in white tea after a certain period of aging [47]. The content of these ketones is higher in LWTs than in PWTs, which may be one of the reasons why LWTs have a higher flowery aroma than PWTs.

Esters, especially those with pleasant aromas, have been identified as key compounds that form the characteristic aroma of white tea [48]. The three esters of VIP > 1 were higher in LWTs than PWTs and mainly displayed “flowery” and “fruit” aromas. Benzyl acetate, as the characteristic aroma component of jasmine tea, is often used in cosmetics and foods to increase the aroma of jasmine and rose [49], which contributes to the flowery aroma of LWTs. Benzyl acetate is present in high volumes in aged dark teas; the reason is that, over time, β-D-glucosidase hydrolyzes the precursors of benzyl alcohol with glucosides to produce benzyl alcohol, which is further converted to benzaldehyde or benzyl acetate [50]. In many types of green tea, the aroma intensity of cis-3-Hexenyl 2-methylbutanoate (green) decreases with increasing storage temperature, which is greatly affected by the storage environment [51].

Alcohols usually have floral, sweet, and fruity aromas, which are the most critical contributor to the formation of white tea aromas [52]. Cedrol (woody, stale), identified as the main aromatic compound in aged white tea and Pu’er tea [53,54], showed the levels of LWTs were twice that of PWTs in almost all groups, which may be one of the reasons why LWTs have a higher woody aroma than PWTs. 2-heptanol has been identified as a key compound in the aroma of coffee and some tropical fruits. Some studies have shown that 2-heptanol has shallow olfactory threshold (OT) content in water (OT = 0.1) and is often described as “fresh fruity”, “lemony”, and “citrus” [55,56]. Terpenes often have spicy, fruity, and woody aromas, which are an essential source of tea aroma [56]. (+)-CUPARENE is a key differential volatile metabolite of roasted green tea at different altitudes [57], and its content is affected by tea grade [58]. Aroma components such as (−)-Calamenene (Herb, spice) are the main contributors to the formation of the irritating camphor fragrance characteristic of eagle tea [59]. Relevant studies have shown that rapid aging technology facilitates the production and accumulation of (−)-Calamenene [35]. In the previous study, volatile fatty acid derivatives and VPBs were significantly enhanced in dark-treated leaves [20]. As a volatile metabolite with VIP value > 1, the content of m-xylene (sweet, flowery) in LWTs was significantly higher than that of PWTs. It is greatly affected by different roasting degrees, and the content in medium-heat tea is significantly higher than that in low-heat tea [60].

The production of various key differential metabolites, such as damasone, 2-decane, benzyl acetate, and (−)-Calamenene, is related to or regulated by the aging process, and the content of these was significantly higher in LWTs than in PWTs. The results showed that long-term understory shade might regulate some metabolic mechanisms in the process of tea aging, and promote the formation of some flavor qualities of aged white tea. Aging gives white tea more pleasant aromas such as rose, sweet, and grassy aromas, and the overall taste is also improved, sweetness, thickness, smoothness, and acidity are enhanced, but bitterness and astringency are weakened [61]. In aged white tea, most ketones (such as 2-decaneone, 2-heptanone, and β-damascatone) and many esters (such as methyl salicylate) are on the rise, accompanied by some newly formed heterocyclic compounds [47], which is consistent with the distribution characteristics of VOCs in LWTs in this study. Understory shade planting may promote the formation of white tea aging flavor, and the specific related metabolic mechanism is a very meaningful research direction, which needs to be studied further.

#### 2.6.2. 32. VOCs with OAV Greater Than 1 in LWTs and PWTs Were Screened for

The aroma of tea is determined by VOCs [62]. The composition of VOCs in white tea is very complex, and most of these compounds are odorless or have a weak odor, so they have little effect on the formation of white tea aroma. The characteristic flavor of the tea is not formed by a simple stacking of VOCs but relies heavily on the interaction between different VOCs, which is closely related not only to the content and aroma type of VOCs, but also to the odor threshold (OT) [63]. Therefore, screening the key active compounds of white tea aroma is essential to systematically reveal their important role in the formation of white tea aroma. Typically, OAVs are used to verify the contribution of active odor compounds and volatile organic compounds, which can well identify key aroma compounds [64,65,66,67,68].

The OAV was calculated for GC-MS test results, and there were 39 VOCs of OVA > 1 (Supplementary Table S3), including 6 alcohols, 2 aromatic hydrocarbons, 1 phenol, eight aldehydes, 5 terpenes, 3 acids, 10 ketones, 3 esters, and 1 heterocyclic compounds. Among them, there were 32 VOCs with OAV greater than 1 in LWTs and PWTs, which are the main contributors to the formation of the characteristic aroma of Xiaobai white tea (Supplementary Table S3). α-Terpineol (HY, TT) and methyl hexanoate (HY, XB, TT) only have OAV values > 1 in individual groups, which may be caused by special woodland environments. Combined with GC-MS-O and aroma recombination experiments, it was found that nalool, (E)-β-damascenone, camphene, 2-hexanone, phenylethyl alcohol, geraniol, and α-terpineol were the main contributors to the aroma of white tea [69], which is basically consistent with the results of this study.

#### 2.6.3. Correlation Analysis between Aroma Profiles and Characteristic Volatile Metabolites of LWTs and PWTs

**Figure 7:**
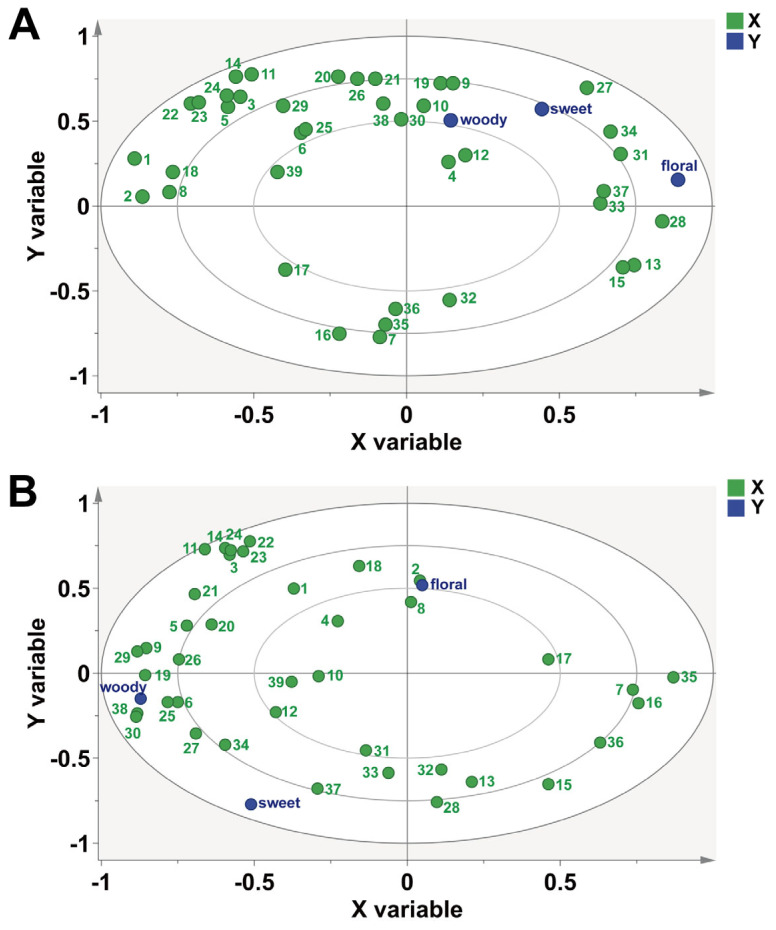
(**A**) PLSR analysis between the aroma properties and the characteristic volatile metabolites (OAVs > 1) of PWTs. X. 39 volatile metabolites (OAVs > 1); Y. aroma properties; (**B**) PLSR analysis between the aroma properties and the characteristic volatile metabolites (OAVs > 1) of LWTs. X. 39 volatile metabolites (OAVs > 1); Y. aroma properties. The serial numbers in the figure correspond to substances in the Supplementary Table S4.

To further confirm the relationships between the volatiles and the aroma properties, PLSR analysis was applied to correlate the important volatiles (OAVs > 1) with the aroma properties (floral, woody, and sweet) in PWTs and LWTs (Figure 7). Geranylacetone, 3,5-OCTADIEN-2-ONE, β-CYCLOCITRAL, and Benzaldehyde were strongly correlated with the floral property. The contents of these substances in LWTs are higher than those in PWTs, which may account for the more pronounced floral aroma of the LWTs. The composition of sweet and woody aroma-related substances was different between PWTs and LWTs. However, LWTs scored higher than PWTs in sweet and woody properties, which may be due to the higher sum of OAV values of understory-related substances.

#### 2.6.4. Four Key Differential VOCs with LWTs and PWTs

**Figure 8:**
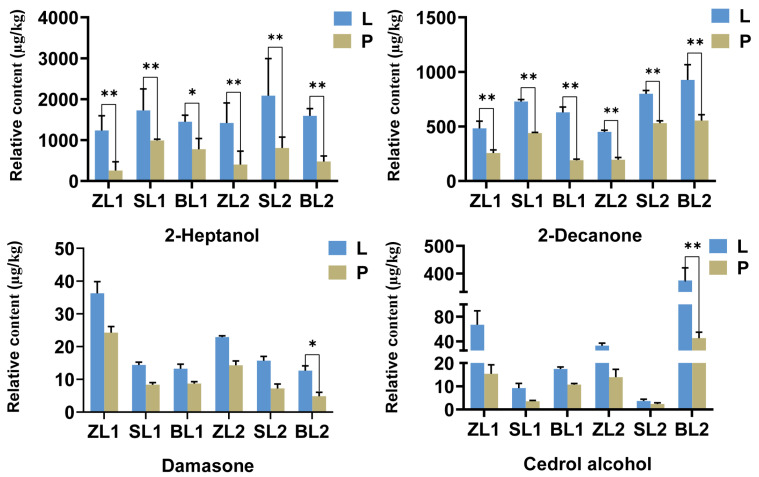
Four key differential volatile metabolites. The symbols * and ** indicate statistical significance at *p* < 0.05 and *p* < 0.01, respectively. Undergrowth-picked was referred to as L, and ordinary-picked was called P.

Key differential volatile metabolites with VIP values greater than 1 are further combined with volatile metabolites with OAV values greater than 1, four substances, 2-heptanol, 2-decane, damasone, and cedar alcohol, were screened as characteristic VOCs that distinguished the difference in flavor quality between LWTs and PWTs, the content of these have a significant difference (Figure 8). It is worth noting that the relative content of cedar alcohol in HX, HY, and other groups is only PWTs (2.38–15.36 μg/kg), LWTs (3.70–66.93 μg/kg), while in TT it is as high as PWTs (45.18 μg/kg) and LWTs (374.91 μg/kg), which showed that different forest environment types had a significant effect on the VOCs content of Xiaobai white tea. It is of great significance to explore the optimal set of Xiaobai white tea forest environment types to improve the quality of LWTs and popularize the planting mode of LWTs.

The analysis of content results showed that cedaryl alcohol and other substances were significantly affected by understory shading. Relevant studies have found, compared with the long-term light of 16 h, 20 h, and 24 h, 12 h light is more conducive to the accumulation of substances contained in white tea and the increase in volatile metabolites such as cedar alcohol (woody, floral), this is consistent with this study [69]. Although 2-heptanol (fresh, sweet, fruity) has been reported in oolong, green, and red raspberry leaf teas [67,68,69], it has been ignored in related studies on white tea. This may be that researchers focus more on compounds with higher content but ignore those with strong odors and low OT. In the recent study, different from previous studies on linalool as the largest contributor to the aroma of white tea, 2-heptanol was found to be the largest contributor to the aroma of GABA white tea for the first time [53]. Damasone (flowery, fried potatoes) has the highest OAV value among the four characteristic volatile metabolites, which is the key contributor to the flowery aroma of LWTs, but there are no studies on shade except for studies showing that its solubility in water is low and affected by storage time.

## 3. Materials and Methods

### 3.1. Chemicals

Hydrochloric acid, disodium hydrogen phosphate, potassium dihydrogen phosphate, ninhydrin, aluminum chloride, folinphenol, sodium bicarbonate, sulfuric acid, sodium carbonate, oxalic acid, ethyl acetate, and n-butanol were purchased from Sinopharm Chemical Reagent Co., Ltd. (Shanghai, China). The plant-soluble sugar content test kit was purchased from Suzhou Keming Biotechnology Co., Ltd. (Suzhou, Jiangsu, China). Sodium chloride, n-hexane (purity > 95%), and 3-hexanone-2,2,4,4-d4 (purity > 95%) were purchased from Sinopharm, Merck, and Sigma-Aldrich (St. Louis, MO, USA), respectively.

### 3.2. Materials

Fresh-leaf samples were taken from *Castanea henryi* understory Xiaobai white tea plantation (ZL), *Cunninghamia lanceolata* understory Xiaobai white tea plantation (SL), *Phyllostachys edulis* understory Xiaobai white tea plantation (BL), and one ordinary Xiaobai white tea plantation (CK) on the same hillside in Zhangdun Township, Jianyang District (118°46′ E, 27°47′ N). According to the five-point sampling method, five tea plants with good growth and no diseases and pests were selected for labeling. The light intensity, air humidity, and air temperature of the marker site were measured by 3415FQF photometric illuminance double radiometer (Spectrum, Stamford, CT, USA) and thermohygrometer every 2 h for 6 consecutive days from 8:00 to 18:00 on 23 April 2022, and the results were averaged. Sampling of fresh leaves of one bud and two leaves was gathered on 28 April 2022, undergrowth-picked was referred to as LF, and ordinary-picked was called PF. Soil samples were taken from 30 cm deep under the canopy of marked tea tree loci, mixed and air-dried for testing.

The Xiaobai white tea samples were produced in Jianyang District, Nanping City, China, from 26 April to 15 May 2023. On 26 April, the fresh leaves of the *Castanea henryi* understory tea plantation and the ordinary tea plantation were collected according to the five-point sampling method with the standard of one bud and two leaves, were withered immediately at 25 °C and 50% relative humidity for 48 h, then dried at 85 °C for 60 min, finally, the *Castanea henryi* understory Xiaobai white tea samples (ZLL1) and the ordinary Xiaobai white tea samples (ZLP1) were obtained. On 30 April, the processing of *Cunninghamia lanceolata* understory Xiaobai white tea samples (SLL1) and ordinary Xiaobai white tea (SLP1) were produced, with the same fresh leaf collecting method and processing technology as above. On 2 May, the processing of *Phyllostachys edulis* understory Xiaobai white tea samples (BLL1) and ordinary Xiaobai white tea samples (BLP1) were produced, with the same fresh leaf collecting method and processing technology as above. On 7 May, 9 May, and 13 May, the *Castanea henryi* understory Xiaobai white tea samples (ZLL2) and ordinary Xiaobai white tea samples (ZLP2), the *Cunninghamia lanceolata* understory Xiaobai white tea samples (SLL2) and ordinary Xiaobai white tea samples (SLP2), and the *Phyllostachys edulis* understory Xiaobai white tea samples (BLL2) and ordinary Xiaobai white tea samples (BLP2) were processed again, respectively. The second rounds of processing of fresh leaf picking method and processing technology as above. Taken together, a total of 12 tea samples were obtained including ZLL1 and ZLP1, SLL1, and SLP1, BLL1 and BLP1, ZLL2, and ZLP2, SLL2, and SLP2, and BLL2 and BLP2, once they were produced. Each sample was composed of three biological replicates, stored at 4 °C, and kept dry for further analysis.

### 3.3. Soil Chemical Analysis

Organic matter was measured using the oxidative thermal potassium dichromate oxidation method [70], the Kjeldahl digestion method was used to extract total nitrogen [71]. Total phosphorus and total potassium were determined using the sodium carbonate method and NaOH melt flamer, respectively [72]. In addition, available nitrogen was measured using the combination pH alkaline hydrolysable method. The hydrochloric acid and flame photometry analyzed the available phosphorus and available potassium, respectively [73]. The pH of soil was measured using the potentiometry [74].

### 3.4. Sensory Evaluation

Tea samples were evaluated and scored by six professional and trained sensory recognition panelists (three females and three males, 30 to 50 years old) from the Fujian Agriculture and Forestry University, who were professional assessors and trained according to the National Professional Standards for Tea Sensory Evaluation (Profession Code: 6–02-06-11, China) and had more than 10 years of descriptive sensory analysis experience with tea. Tea infusions were prepared according to national standards (GB/T 23776-2018) [75]: each tea sample (3 g) was brewed with 150 mL boiling water for 5 min and discarded, after which tea infusions labelled with a three-digit code were presented to each panelist in a randomized order. Then, the intensity values (0–10), taste descriptors (smoothness, sweetness, thickness, astringency, and umami), and aroma descriptors (floral, sweet, and woody) of each sample infusion were subjected to a sensory test by the six panelists. A scale from 0 to 10 (where 0 was none or no perception and 10 was extremely strong) as described in a previous study was used to symbolize intensity values [76].

### 3.5. Macro-Composition Quantification

Water extracts were measured using the suggested protocol from the Chinese National Standard, GB/T 8305-2013) [77], free amino acids were measured using the suggested protocol from the Chinese National Standard (GB/T 8314-2013) [78], tea polyphenols were measured using the suggested protocol from the Chinese National Standard (GB/T 8313-2018) with minor changes [79], caffeine was quantified using the UV spectrophotometric method based on the National Standard of China (GB/T 8312-2013) [80], soluble sugars were quantified using the plant-soluble sugar content test kit (Grace Biotechnology Co., Ltd., Suzhou, China) according to the manufacturer’s instructions.

Quantification of total flavonoids content was according to an ultraviolet–visible spectrometry method reported by Ma et al. [81]. Briefly, the reaction contained 0.25 mL of the 70% (*v*/*v*) methanol extract or rutin standard solution and 1.25 mL of distilled water, followed by the addition of 0.75 mL 5% (*m*/*v*) sodium nitrite solution was mixed and rested for 5 min. Then, 0.15 mL of 10% (*m*/*v*) aluminum chloride solution was added and stood for 5 min before 0.5 mL of 1 M sodium hydroxide was added. The absorbance of the mixture was measured at 510 nm. The data were calculated using rutin as total flavonoids.

The total content of TBs, TFs, and TRs was determined using a previously discussed method [40]. Briefly, add 125 mL of boiling water to 3 g of tea powder, bathe in water for 10 min, filter, and cool to room temperature. The amount of 50 mL of ethyl acetate was pipetted in 50 mL of tea filtrate and shaken for 5 min, and the layers were separated after equilibration. A 0.8 mL portion of the ethyl acetate layer was pipetted in 10 mL test tubes and fixed volume with 95% ethanol (solution A). The 15 mL of NaHCO_3_ solution (2.5%) was pipetted in 15 mL of the ethyl acetate layer and shaken for 30 s, testing to layering. A 1.6 mL ethyl acetate layer was pipetted in 10 mL test tubes and fixed volume with 95% ethanol (solution C). A 0.8 mL sample of portions of the aqueous layer was diluted to 10 mL with 2.4 mL distilled water, 0.8 mL saturated oxalic acid solution (10.2%, *m*/*v*), and fixed volume with 95% ethanol (solution D). A 25 mL amount of tea filtrate was pipetted and mixed with 25 mL of butyl alcohol. After shaking for 3 min, the layers were separated after equilibration. A 0.4 mL sample of the aqueous layer (second) was made to a volume of 10 mL with 0.4 mL of the saturated oxalic acid solution, 1.2 mL of distilled water, and 95% ethanol (solution B). The absorbance of solutions A, B, C, and D at 380 nm was measured spectrophotometrically using 95% ethanol as a blank. The results were calculated using the following formula:

(1) $${TF}_{S}=\frac{E_{c}\times 2.25}{dried\;weight\;(\%)}\times 100\%$$

(2) $${TR}_{S}=\frac{7.06\times \left(2EA+2ED-EC-2EB\right)}{dried\;weight\;(\%)}\times 100\%$$

(3) $${TB}_{S}=\frac{2EB\times 7.06}{dride\;weight\;(\%)}\times 100\%$$

### 3.6. Identification and Analysis of Volatiles

Volatiles were extracted from the samples using the headspace solid-phase microextraction (HS-SPME) method. After sampling, volatile analysis was performed using an Agilent Model 8890 GC equipped with a 30 m × 0.25 mm × 0.25 µm DB-5MS (5% phenyl-polymethylsiloxane) capillary column and a 7000 D mass spectrometer (Agilent, Santa Clara, CA, USA). The procedures of HS-SPME and gas chromatography–mass spectrometry (GC-MS) analysis were performed, as previously described [40], with minor modifications. The temperature was programmed at 40 °C for 3.5 min, increasing to 100 °C at a rate of 10 °C/min, then to 180 °C at a rate of 7 °C/min, and finally to 280 °C at a rate of 25 °C/min and hold for 5 min. After that, VOCs were identified using the 7000 D MS (Agilent). The MS spectrometer operates in electron shock mode with an electron energy of 70 eV and a scanning range of 50–500 m/z. The temperatures of the ion source, quadrupole, and transfer line were set at 230, 150, and 280 °C, respectively. The more detailed GC-MS acquisition conditions are listed in Supplementary Table S5. The volatile peaks were identified by matching the National Institute of Standards and Technology (NIST) mass spectral database and retention index (RI, determined by n-alkane C7–C40). The chemical structure, name, and aroma of volatile organic compounds are from PubChem (https://pubchem.ncbi.nlm.nih.gov, accessed on 10 June 2023) and Good Scents Company Information System (http://www.thegoodscentscompany.com, accessed on 10 June 2023). The internal standard used was saturated NaCl solution (10 µL, 50 µg/mL), and the relative content of each volatile compound was calculated using the following formula:

(4) $$C_{i}=\frac{\frac{A_{i}}{A_{is}\;\times \;m_{is}}}{m_{i}}$$

where *Ci* is the mass concentration of each component (μg kg^−1^), m_is_ is the mass of the internal standard (μg), *A_i_*, and *A_is_* are the chromato-graphic peak area of each component and internal standard, respectively, and mi is the mass of the sample powder (kg).

OAV was calculated by dividing the calculated concentration of the volatile compound by its odor threshold in water and was used to evaluate the contributions of the volatile compounds to the aroma of tea samples. It is generally believed that OAV ≥ 1 of volatile compounds contributes to the flavor of samples [82]. The calculation formula for OAV value is:

(5) $${OAV}_{i}=\frac{C_{i}}{{OT}_{i}}$$

Note: *Ci* (μg/kg) was the content of the volatile compounds; *O_Ti_* (μg/kg) was the aroma threshold of volatile components in water.

### 3.7. Statistical Analysis

All analyses were repeated three times. The statistical significance among the different flavors of Xiaobai white tea was determined by one-way analysis of variance (ANOVA) and Duncan’s multiple range test using SPSS (Version 25.0, Armonk, NY, USA) [83]. TBtools (https://github.com/CJ-Chen/TBtools, accessed on 10 July 2023) was used for heat-map and hierarchical cluster analysis of the macro-Composition and volatile components. A radar plot was drawn using Origin (v2021, OriginLab Corporation, Northampton, MA, USA) [84]. OPLS-DA was performed using SIMCA software (version 13.0; Umetrics, Umea, Sweden) [85].

## 4. Conclusions

In this study, the macro-composition and volatile compounds of LWTs and PWTs were determined and analyzed by biochemical composition determination, widely targeted volatilities (WTV) analysis, multivariate statistical analysis, and odor activity value (OAV) analysis. The results show that understory planting improves the growth environment of tea plants, promotes the formation of the components of fresh-leaf, and provides a good material basis for the processing of dry tea. The free amino acids, TFs, TRs, water-extractable substances, tea polyphenols, 2-heptanol, 2-decane, damasone, and cedar alcohol were the key macro-composition and volatile compounds to distinguish LWTs and PWTs. This study will help to improve the quality of white tea and promote the application of the planting mode of Xiaobai white tea under forest.

## Figures and Tables

**Table 1:** Fresh-leaf samples information.

Identifier	Source of Fresh Leaves	Picking Date	Picking Standards	Identifier
CK	ordinary tea plantation	25 April 2023	one bud and two leaves	CK-1 to 3
ZL	*Castanea henryi* understory tea plantation			ZL-1 to 3
SL	*Cunninghamia lanceolata* understory tea plantation			SL-1 to 3
BL	*Phyllostachys edulis* understory tea plantation			BL-1 to 3

**Table 2:** Xiaobai white tea samples information.

Identifier	Source of Fresh Leaves	Production Date	Picking Standards	Identifier
ZL1	*Castanea henryi* understory tea plantation	26–28 April 2023	one bud and two leaves	ZLL1-1 to 3
	ordinary tea plantation			ZLP1-1 to 3
SL1	*Cunninghamia lanceolata* understory tea plantation	30 April–2 May 2023		SLL1-1 to 3
	ordinary tea plantation			SLP1-1 to 3
BL1	*Phyllostachys edulis understory tea plantation*	2–5 May 2023		BLL1-1 to 3
	ordinary tea plantation			BLP1-1 to 3
ZL2	*Castanea henryi* understory tea plantation	7–9 May 2023		ZLL2-1 to 3
	ordinary tea plantation			ZLP2-1 to 3
SL2	*Cunninghamia lanceolata* understory tea plantation	9–12 May 2023		SLL2-1 to 3
	ordinary tea plantation			SLP2-1 to 3
BL2	*Phyllostachys edulis* understory tea plantation	13–15 May 2023		BLL2-1 to 3
	ordinary tea plantation			BLP2-1 to 3

## Data Availability

Data are contained within the article or Supplementary Materials.

## Supplementary Materials

The following supporting information can be downloaded at: https://www.mdpi.com/article/10.3390/plants12244102/s1, Figure S1: Total ion chromatogram of mixed sample mass spectrometry analysis for quality control; Figure S2: Scatter plot of volatile components of OPLS-DA scores for six sets of samples (A), (C), (E), (G), (I), and (K); OPLS-DA cross-validation results of volatile components in six groups of samples (B), (D), (F), (H), (J), and (L). Table S1: The qualitative results for the VOCs in LWTs and PWT; Table S2: VIP and FC values of the 13 volatile components; Table S3: The VOCs of OVA is greater than 1 in all samples; Table S4: The serial number in the PLSR analysis corresponds to the VOCs; Table S5: The headspace solid-phase micro extraction (HS-SPME) and gas chromatography-mass spectrometry (GC-MS) conditions.

## References

[1] Mohotti A.J., Lawlor D.W. (2002). Diurnal variation of photosynthesis and photoinhibition in tea: Effects of irradiance and nitrogen supply during growth in the field. J. Exp. Bot..

[2] Chen Q., Shi J., Mu B., Chen Z., Dai W., Lin Z. (2020). Metabolomics combined with proteomics provides a novel interpretation of the changes in nonvolatile compounds during white tea processing. Food Chem..

[3] Ran W., Li Q., Hu X., Zhang D., Yu Z., Chen Y., Wang M., Ni D. (2023). Comprehensive analysis of environmental factors on the quality of tea (*Camellia sinensis* var. *sinensis*) fresh leaves. Sci. Hortic..

[4] Shao C., Deng Z., Liu J., Li Y., Zhang C., Yao S., Zuo H., Shi Y., Yuan S., Qin L. (2022). Effects of Preharvest Shading on Dynamic Changes in Metabolites, Gene Expression, and Enzyme Activity of Three Tea Types during Processing. J. Agric. Food Chem..

[5] Santosh K.C., Long L., Zhang Q., Ni K., Ma L., Ruan J. (2022). Effect of Interactions between Phosphorus and Light Intensity on Metabolite Compositions in Tea Cultivar Longjing43. Int. J. Mol. Sci..

[6] Wang M., Yang J., Li J., Zhou X., Xiao Y., Liao Y., Tang J., Dong F., Zeng L. (2022). Effects of temperature and light on quality-related metabolites in tea [*Camellia sinensis* (L.) *Kuntze*] leaves. Food Res. Int..

[7] Wang Y., Zhang Q., Li J., Lin S., Jia X., Zhang Q., Ye J., Wang H., Wu Z. (2023). Study on the Effect of pH on Rhizosphere Soil Fertility and the Aroma Quality of Tea Trees and Their Interactions. Agriculture.

[8] Ye J., Chen X., Liu G., Jia X., Zhang Q., Zhu C., Wang Y., Wang H.B. (2021). Effect of tea soil acidification on the diversity and function of fungi community. J. Appl. Bot. Food Qual..

[9] Tang S., Zhou J., Pan W., Sun T., Liu M., Tang R., Li Z., Ma Q., Wu L. (2023). Effects of combined application of nitrogen, phosphorus, and potassium fertilizers on tea (*Camellia sinensis*) growth and fungal community. Appl. Soil. Ecol..

[10] Chen P., Lin S., Liu C., Su Y., Cheng H., Shiau J., Chen I. (2015). Correlation between nitrogen application to tea flushes and quality of green and black teas. Sci. Hortic..

[11] Lin Z., Qi Y., Chen R., Zhang F., Chen L. (2012). Effects of phosphorus supply on the quality of green tea. Food Chem..

[12] Artru S., Garré S., Dupraz C., Hiel M., Blitz-Frayret C., Lassois L. (2017). Impact of spatio-temporal shade dynamics on wheat growth and yield, perspectives for temperate agroforestry. Eur. J. Agron..

[13] Quinkenstein A., Wöllecke J., Böhm C., Grünewald H., Freese D., Schneider B.U., Hüttl R.F. (2009). Ecological benefits of the alley cropping agroforestry system in sensitive regions of Europe. Environ. Sci. Policy.

[14] Albrecht A., Kandji S.T. (2003). Carbon sequestration in tropical agroforestry systems. Agric. Ecosyst. Environ..

[15] Torralba M., Fagerholm N., Burgess P.J., Moreno G., Plieninger T. (2016). Do European agroforestry systems enhance biodiversity and ecosystem services? A meta-analysis. Agric. Ecosyst. Environ..

[16] Wang Y., Mo Y.R., Tan J., Wu L.X., Pan Y., Chen X.D. (2022). Effects of growing *Coptis chinensis* Franch in the natural understory vs. under a manmade scaffold on its growth, alkaloid contents, and rhizosphere soil microenvironment. Peer J..

[17] Huang Z., Shen Z., Liu C., Shi H., He S., Long G., Deng W., Yang J.L., Fan W. (2022). Characteristics of heavy metal accumulation and risk assessment in understory Panax notoginseng planting system. Environ. Geochem. Health.

[18] Sgarbossa J., Elli E.F., Schwerz F., Nardini C., de Cristo E., de Oliveira D., Caron B.O. (2020). Morphology, growth and yield of black oats cultivated in agroforestry systems in southern Brazil. Agric. Syst..

[19] Liu J., Yuan D., Si H., Pang X., Tang X., Yang J. (2013). Effects of Shading on Ingredients of Tea Shoots in Different Seasons. Southwest China J. Agric. Sci..

[20] Yang Z., Kobayashi E., Katsuno T., Asanuma T., Fujimori T., Ishikawa T., Tomomura M., Mochizuki K., Watase T., Nakamura Y. (2012). Characterisation of volatile and non-volatile metabolites in etiolated leaves of tea (*Camellia sinensis*) plants in the dark. Food Chem..

[21] Shu J., Xu Y. (1990). Microscopic and ultrastructural observations of tea tree leaves. China Tea.

[22] Fang Q., Jin J., Zhu Y., Ye J., Dong J., Liang Y. (2021). Effect of shade treatmen and harvest season on the taste and aroma-related components in steamed green tea. J. Tea.

[23] Yang X., Leng Y., Zhou Z., Shang H., Ni K., Ma L., Yi X., Cai Y., Ji L., Ruan J. (2022). Ecological management model for the improvement of soil fertility through the regulation of rare microbial taxa in tea (*Camellia sinensis* L.) plantation soils. J. Environ. Manag..

[24] Duan X. (2008). Practical New Technology for Quality Tea Production.

[25] Li X., Ahammed G.J., Li Z., Zhang L., Wei J., Shen C., Yan P., Zhang L., Han W. (2016). Brassinosteroids Improve Quality of Summer Tea (*Camellia sinensis* L.) by Balancing Biosynthesis of Polyphenols and Amino Acids. Front. Plant Sci..

[26] Tongsiri P., Tseng W., Shen Y., Lai H. (2020). Comparison of Soil Properties and Organic Components in Infusions According to Different Aerial Appearances of Tea Plantations in Central Taiwan. Sustainability.

[27] Wen B., Zhang X., Ren S., Duan Y., Zhang Y., Zhu X., Wang Y. (2020). Characteristics of soil nutrients, heavy metals and tea quality in different intercropping patterns. Agrofor. Syst..

[28] De Costa W.A.J.M., Surenthran P., Attanayake K.B. (2005). Tree-crop interactions in hedgerow intercropping with different tree species and tea in Sri Lanka: 2. Soil and plant nutrients. Agrofor. Syst..

[29] Zhang G., Chu X., Zhu H., Zou D., Li L., Du L. (2021). The Response of Soil Nutrients and Microbial Community Structures in Long-Term Tea Plantations and Diverse Agroforestry Intercropping Systems. Sustainability.

[30] Huang L., Xu K., Zhou C., Shi B., Tian C., Lu L., Guo Y. (2022). Quality Differences of Zhenghe White Tea from Different Altitudes. Food Sci..

[31] Wang S., Yin K., Liang G. (2002). Multivariate correlation analysis of leaf anatomy and quality traits of tea plants. J. Tea Bus..

[32] Chen Y., Fu X., Mei X., Zhou Y., Cheng S., Zeng L., Dong F., Yang Z. (2017). Proteolysis of chloroplast proteins is responsible for accumulation of free amino acids in dark-treated tea (*Camellia sinensis*) leaves. J. Proteom..

[33] Lu A., Ye Y., Liao X., Mao S., Yan J., Yang L., Zhang Y., Tong H. (2019). Effects of Shading on Main Nitrogen Compounds in Fresh Leaves of Tea Plant. Chin. Agric. Sci. Bull..

[34] Fu H., Li H., Yin P., Mei H., Li J., Zhou P., Wang Y., Ma Q., Jeyaraj A., Thangaraj K. (2021). Integrated Application of Rapeseed Cake and Green Manure Enhances Soil Nutrients and Microbial Communities in Tea Garden Soil. Sustainability.

[35] Penn C.J., Camberato J.J. (2019). A Critical Review on Soil Chemical Processes that Control How Soil pH Affects Phosphorus Availability to Plants. Agriculture.

[36] Shao C., Jiao H., Chen J., Zhang C., Liu J., Chen J., Li Y., Huang J., Yang B., Liu Z. (2022). Carbon and Nitrogen Metabolism Are Jointly Regulated During Shading in Roots and Leaves of Camellia Sinensis. Front. Plant Sci..

[37] Du Y., Zhao Q., Chen L., Yao X., Zhang W., Zhang B., Xie F. (2020). Effect of drought stress on sugar metabolism in leaves and roots of soybean seedlings. Plant Physiol. Biochem..

[38] Gong X., Li L., Qin L., Huang Y., Ye Y., Wang M., Wang Y., Xu Y., Luo F., Mei H. (2022). Targeted Metabolomics Reveals Impact of N Application on Accumulation of Amino Acids, Flavonoids and Phytohormones in Tea Shoots under Soil Nutrition Deficiency Stress. Forests.

[39] Lin Z., Chen C., Zhong Q., Ruan Q., Chen Z., You X., Shan R., Li X. (2021). The GC-TOF/MS-based Metabolomic analysis reveals altered metabolic profiles in nitrogen-deficient leaves and roots of tea plants (*Camellia sinensis*). BMC Plant Biol..

[40] Zhang C., Zhou C., Xu K., Tian C., Zhang M., Lu L., Zhu C., Lai Z., Guo Y. (2022). A Comprehensive Investigation of Macro-Composition and Volatile Compounds in Spring-Picked and Autumn-Picked White Tea. Foods.

[41] Ho C., Zheng X., Li S. (2015). Tea aroma formation. Food Sci. Hum. Well..

[42] Liu Z., Chen F., Sun J., Ni L. (2021). Dynamic changes of volatile and phenolic components during the whole manufacturing process of Wuyi Rock tea (Rougui). Food Chem..

[43] Lv S., Wu Y., Li C., Xu Y., Liu L., Meng Q. (2014). Comparative Analysis of Pu-erh Tea and Fuzhuan Tea by Fully Automatic Headspace Solid-Phase Microextraction Coupled with Gas Chromatography-Mass Spectrometry and Chemometric Methods. J. Agric. Food Chem..

[44] Ni Z., Zhou Z., Liu B., Gao F., Li L., Wu Q., Deng H., Sun Y. (2021). Study on Flavor Components in “Cloud-Mist Mountain Tea” Green Tea. Sci. Technol. Food Ind..

[45] Wang H. (2017). Study on the Characteristic Aroma Components of Keemun Black Tea. Master’s Thesis.

[46] Shen S., Wu H., Li T., Sun H., Wang Y., Ning J. (2023). Formation of aroma characteristics driven by volatile components during long-term storage of An tea. Food Chem..

[47] Qi D., Miao A., Cao J., Wang W., Chen W., Pang S., He X., Ma C. (2018). Study on the effects of rapid aging technology on the aroma quality of white tea using GC–MS combined with chemometrics: In comparison with natural aged and fresh white tea. Food Chem..

[48] Wang L., Cai L., Lin Z., Zhong Q., Lv H., Tan J., Guo L. (2010). Analysis of Aroma Compounds in White Tea Using Headspace Solid-phase Micro-extraction and GC-MS. J. Tea Sci..

[49] Lin J., Chen Y., Zhang P., Ren M., Xu H., Wang X. (2013). A novel quality evaluation index and strategies to identify scenting quality of jasmine tea based on headspace volatiles analysis. Food Sci. Biotechnol..

[50] Nie C., Du X., Gao Y., He L., Wang C., Zhang X., Zhong X. (2019). Comparison of different aroma-active compounds of Sichuan Dark brick tea (*Camellia sinensis*) and Sichuan Fuzhuan brick tea using gas chromatography–mass spectrometry (GC–MS) and aroma descriptive profile tests. Eur. Food Res. Technol. = Z. Fur Lebensm. Unters. Forschung. A.

[51] Dai Q., Jin H., Gao J., Ning J., Yang X., Xia T. (2019). Investigating Volatile Compounds’ Contributions to the Stale Odor of Green Tea. Int. J. Food Sci. Technol..

[52] Li Y., Wu T., Deng X., Tian D., Ma C., Wang X., Li Y., Zhou H. (2023). Characteristic aroma compounds in naturally withered and combined withered γ-aminobutyric acid white tea revealed by HS-SPME-GC-MS and relative odor activity value. LWT.

[53] Chen Z., Li P., Chen X., Yang Y., He P., Tu Y. (2020). Effect of Compressed Processing on the Aroma of Aged White Tea. Sci. Technol. Food Ind..

[54] Wang Q., Chen D. (2012). Research on characteristic aroma material in Guangdong Chenxiang tea. Guangdong Agric. Sci..

[55] El Hadi M.A., Zhang F., Wu F., Zhou C., Tao J. (2013). Advances in Fruit Aroma Volatile Research. Molecules.

[56] Zhou Z., Wang X., Luo Z., Fan G., Tian C., Tang Q., Pan S. (2012). Identification and Analysis of Free and O-Glycoside-Bound Volatile Components in Wild *Rosa roxburghii* Juice. Food Sci..

[57] Zhang Y., Zhu Y., Lv H., Huang H., Shao C., Peng J., Lin Z. (2022). Quality composition analysis of roasted green tea at different altitudes. Food Sci..

[58] Yin X., Fu W., Chen Y., Zhou R., Sun W., Ding B., Peng X., Gu H. (2022). GC-MS-based untargeted metabolomics reveals the key volatile organic compounds for discriminating grades of Yichang big-leaf green tea. LWT.

[59] Zhang L., Wang J., Luo L., Zeng L. (2019). Analysis of characteristic aroma components of eagle tea. Food Sci..

[60] Xu X. (2020). Study on the Difference of Roasting Degree, Grade and Regional Quality of Wuyi Cinnamon Tea Based on Metabolomics. Master’s Thesis.

[61] Fan F., Huang C., Tong Y., Guo H., Zhou S., Ye J., Gong S. (2021). Widely targeted metabolomics analysis of white peony teas with different storage time and association with sensory attributes. Food Chem..

[62] Xue J., Guo G., Liu P., Chen L., Wang W., Zhang J., Yin J., Ni D., Engelhardt U., Jiang H. (2022). Identification of aroma-active compounds responsible for the floral and sweet odors of Congou black teas using gas chromatography–mass spectrometry/olfactometry, odor activity value, and chemometrics. J. Sci. Food Agric..

[63] Guo X., Schwab W., Ho C., Song C., Wan X. (2022). Characterization of the aroma profiles of oolong tea made from three tea cultivars by both GC–MS and GC-IMS. Food Chem..

[64] Huang Y., Wan J., Wang Z., Sun M., Feng T., Ho C., Song S. (2022). Variation of Volatile Compounds and Corresponding Aroma Profiles in Chinese Steamed Bread by Various Yeast Species Fermented at Different Times. J. Agric. Food Chem..

[65] Huang G., Li Y., Deng X., Su D., Shen Y., Li Y., Zhou H. (2022). Analysis of aroma compounds of Yunnan white tea by four drying methods. Sci. Technol. Food Ind..

[66] Yuan Q., Tu M., Gao P., Hu C., He D. (2020). Comparative Analysis of Rapeseed Oils Prepared by Three Different Methods. J. Oleo Sci..

[67] Wang W., Huang X., Lin Y., Tang R., Guo Y. (2019). Analysis of volatile compounds in Guanyin tea stem. Trop. Crops.

[68] Yu S. (2017). Multi-Wavelength LED White Tea Quality Control and Its Production Line Research. Master’s Thesis.

[69] Ni H., Jiang Q., Zhang T., Huang G., Li L., Chen F. (2020). Characterization of the Aroma of an Instant White Tea Dried by Freeze Drying. Molecules.

[70] Roper W.R., Robarge W.P., Osmond D.L., Heitman J.L. (2019). Comparing Four Methods of Measuring Soil Organic Matter in North Carolina Soils. Soil. Sci. Soc. Am. J..

[71] Keeney D.R., Nelson D.W. (2015). Methods of Soil Analysis. Part 2. Chemical and Microbiological Properties.

[72] Lin W., Lin M., Zhou H., Wu H., Li Z., Lin W. (2019). The effects of chemical and organic fertilizer usage on rhizosphere soil in tea orchards. PLoS ONE.

[73] Mózo B.S., Benites A.C., Sukma M., Paul M.M. (2016). Handbook of Soil Analysis: Mineralogical, Organic and Inorganic Methods.

[74] (2018). Soil—Determination of pH—Potentiometry.

[75] (2018). Methodology for Sensory Evaluation of Tea.

[76] Guo X., Ho C., Wan X., Zhu H., Liu Q., Wen Z. (2021). Changes of volatile compounds and odor profiles in Wuyi rock tea during processing. Food Chem..

[77] (2013). Tea—Determination of Water Extracts Content.

[78] (2013). Tea—Determination of Free Amino Acids Content.

[79] (2018). Determination of Total Polyphenols and Catechins Content in Tea.

[80] (2013). Tea—Determination of Caffeine Content.

[81] Ma Z., Li S., Zhang M., Jiang S., Xiao Y. (2010). Light Intensity Affects Growth, Photosynthetic Capability, and Total Flavonoid Accumulation of Anoectochilus Plants. HortScience.

[82] Li K., Wang J., Li Y., Li Q. (2019). Characteristic aroma components of red pitaya wine. Food Ferment. Ind..

[83] IBM Corp. (2017). IBM SPSS Statistics for Windows.

[84] (2021). Origin(Pro).

[85] (2012). SIMCA.

