# Supplementary material for: Analysis of Characteristics in the Macro-Composition and Volatile Compounds of Understory Xiaobai White Tea
Source: Plants (Basel). 2023 Dec 7;12(24):4102. doi: 10.3390/plants12244102 (PMC10747399; doi:10.3390/plants12244102)
Supplement: Supplementary file 1 [file plants-12-04102-s001.zip › Supplementary Figures.pdf]

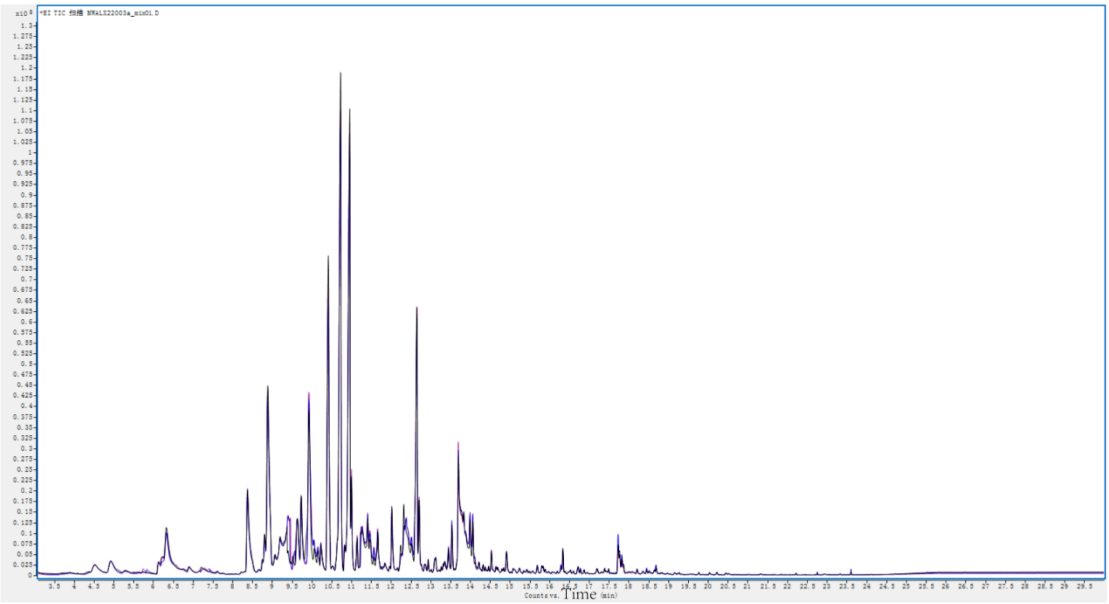

Figure S1. Total ion chromatogram of mixed sample mass spectrometry analysis for quality control

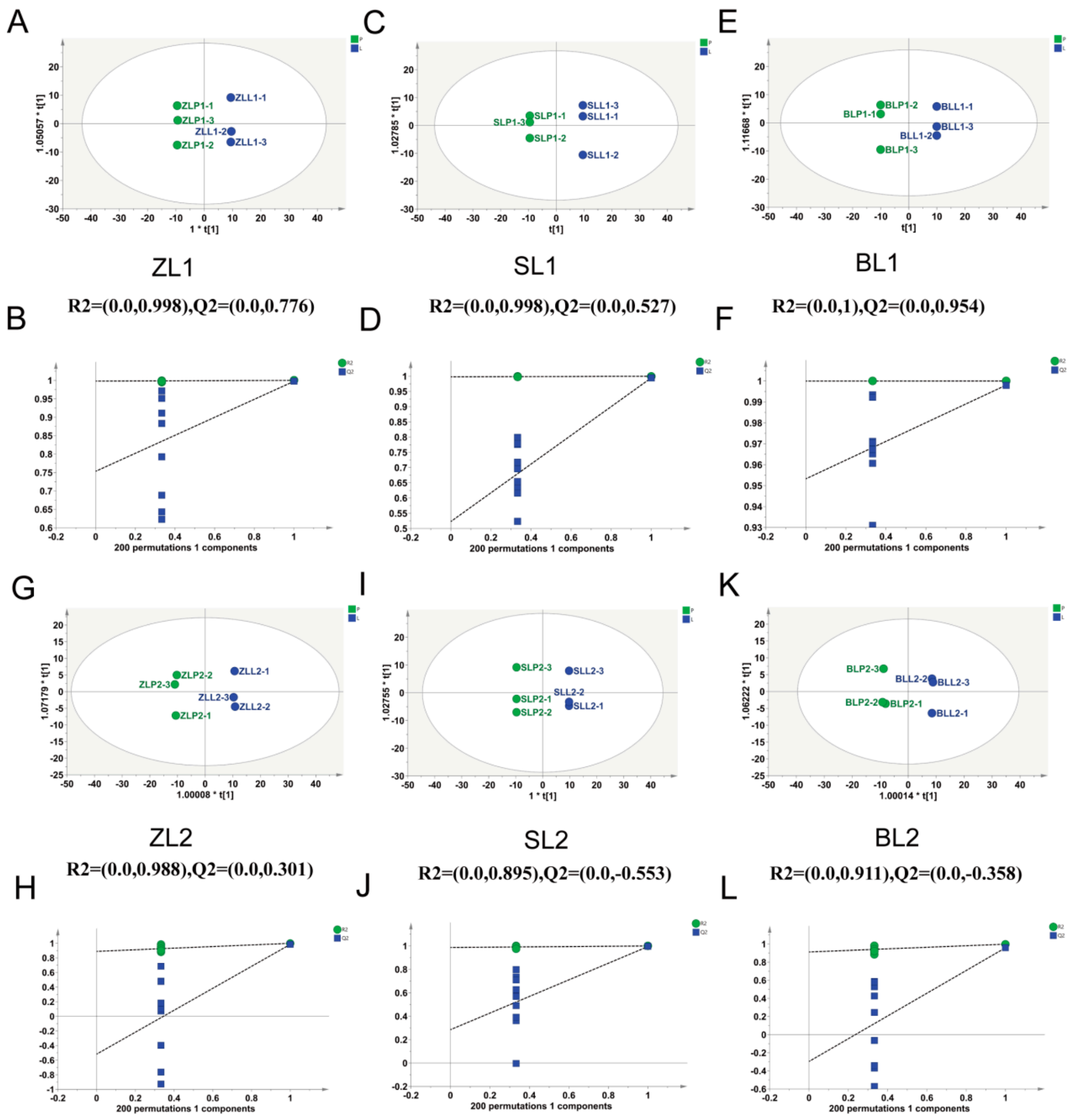

Figure S2. Scatter plot of volatile components of OPLS-DA scores for six sets of samples (A), (C), (E), (G), (I) and (K); OPLS-DA cross-validation results of volatile components in six groups of samples (B), (D), (F), (H), (J) and (L).
